# Associations of Mitochondrial Fatty Acid Oxidation with Body Fat in Premenopausal Women

**DOI:** 10.1155/2017/7832057

**Published:** 2017-10-24

**Authors:** Jonathan L. Warren, Barbara A. Gower, Gary R. Hunter, Samuel T. Windham, Douglas R. Moellering, Gordon Fisher

**Affiliations:** ^1^Department of Nutrition Sciences, University of Alabama at Birmingham, 1720 2nd Avenue South, Birmingham, AL 35294, USA; ^2^Department of Human Studies, University of Alabama at Birmingham, 1720 2nd Avenue South, Birmingham, AL 35294, USA; ^3^Department of Surgery, University of Alabama at Birmingham, 1720 2nd Avenue South, Birmingham, AL 35294, USA

## Abstract

Higher* in vivo* fatty acid (FA) oxidation rates have been reported in obese individuals compared to lean counterparts; however whether this reflects a shift in substrate-specific oxidative capacity at the level of the skeletal muscle mitochondria has not been examined. The purpose of this study was to test the hypothesis that in situ measures of skeletal muscle mitochondria FA oxidation would be positively associated with total body fat. Participants were 38 premenopausal women (BMI = 26.5 ± 4.3 kg/m^2^). Total and regional fat were assessed by dual-energy X-ray absorptiometry (DXA). Mitochondrial FA oxidation was assessed in permeabilized myofibers using high-resolution respirometry and a palmitoyl carnitine substrate. We found positive associations of total fat mass with State 3 (ADP-stimulated respiration) (*r* = 0.379, *p* < 0.05) and the respiratory control ratio (RCR, measure of mitochondrial coupling) (*r* = 0.348, *p* < 0.05). When participants were dichotomized by high or low body fat percent, participants with high total body fat displayed a higher RCR compared to those with low body fat (*p* < 0.05). There were no associations between any measure of regional fat and mitochondrial FA oxidation independent of total fat mass. In conclusion, greater FA oxidation in obesity may reflect molecular processes that enhance FA oxidation capacity at the mitochondrial level.

## 1. Introduction

A number of studies have shown enhanced FA oxidation in obese individuals compared to lean counterparts using* in vivo* approaches to assess FA oxidation capacity and anthropometric measures of obesity [1–3]. Whether this observation reflects a shift in substrate-specific oxidative capacity at the level of the skeletal muscle mitochondria has not been examined.

The process of FA oxidation is dependent on a number of factors that cannot be disentangled using* in vivo* approaches. FA must first be transported into cells via fatty acid translocase (CD36), followed by FA transport into the mitochondria via carnitine palmitoyltransferase 1B (CPT1B), and ultimately *β*-oxidation and oxidative phosphorylation within the mitochondria [4]. Potential alterations at any of these regulatory steps cannot be identified by* in vivo* measures of FA oxidation. In contrast, studying FA oxidation in skeletal muscle mitochondria in situ can assist in isolating the level at which changes or differences in whole-body fat oxidation occur. Thus, these in situ measures can be used to examine whether mechanisms promoting increased whole-body FA oxidation in obese individuals are reflected in changes in mitochondrial capacity.

Mitochondrial capacity is modifiable in order to respond to changes in both acute and chronic metabolic demands [5]. In the context of the chronic metabolic condition of obesity, increases in mitochondrial FA oxidative capacity may be mediated in part by peroxisome proliferator-activated receptor *γ* coactivator 1*α* (PGC1*α*), a protein that promotes the transcription of genes associated with FA metabolism and is upregulated by a high-fat diet and obesity in rodent models [6, 7]. Elevated FA oxidation can also interfere with the oxidation of glucose [8], which may lead to a continued reliance on FA substrate [9]. Determining whether enhanced FA oxidation observed in obese humans is an acquired phenotype regulated in part by the mitochondria may ultimately lead to novel mechanisms for prevention or treatment of obesity and cardiometabolic diseases.

The overall purpose of this study was to test the hypothesis that in situ measures of skeletal muscle mitochondria FA oxidation would be positively associated with total body fat in a sample of premenopausal women. We hypothesized that total fat mass would be positively associated with skeletal muscle mitochondrial FA oxidation. Additionally, we examined whether regional fat distribution patterns were independent predictors of mitochondrial FA oxidation.

## 2. Methods

### 2.1. Participants

Participants were 38 premenopausal female participants self-identifying as European-American (EA) or African-American (AA). Inclusion criteria were as follows: BMI of 18.5–35.0 kg/m^2^, being sedentary (<30 minutes of structured activity per week), being nondiabetic, not using medication known to affect body composition or metabolism including oral contraceptives. All testing was conducted during the first 10 days of the follicular phase of the menstrual cycle. All participants provided informed consent. This study was approved by the Institutional Review Board at the University of Alabama at Birmingham.

### 2.2. Total and Regional Fat Quantification

Body composition was assessed by dual-energy X-ray absorptiometry (DXA) using a Lunar iDXA densitometer with enCORE™ software (GE-Lunar Corporation, Madison, WI). Participants were scanned in light clothing lying supine with arms at their sides. The scans were assessed for total body fat-free mass (kg), total body fat mass (kg), total tissue fat percentage (%), leg fat mass (kg), android region fat mass (kg), and visceral adipose tissue (VAT) volume (cm^3^).

### 2.3. In Vivo Substrate Utilization

Resting respiratory quotient (RQ) was obtained in a subset of 17 participants. Following an overnight fast, resting RQ was assessed by indirect calorimetry (Vmax ENCORE 29N Systems, SensorMedics Corporation, Yorba Linda, CA) [10]. Each measure was obtained following a 30 min rest period in a supine position. A canopy hood was placed over the head of the participant in order to collect expired air. Oxygen consumption (VO_2_) and carbon dioxide production (VCO_2_) were recorded each minute for 30 min. The average VO_2_ and VCO_2_ measured during a steady state identified by the software were calculated, and resting RQ was recorded as the ratio of VCO_2_/VO_2_. A lower RQ reflects relatively greater use of fatty acids as a substrate.

### 2.4. Laboratory Analyses

Sera were analyzed by the Core Laboratory of the UAB Nutrition Obesity Research Center, Diabetes Research Center, and Center for Clinical and Translational Science. Fasting glucose, total cholesterol, HDL cholesterol, triglycerides, and circulating free FA were measured using a SIRRUS analyzer (Stanbio Laboratory, Boerne, TX). Fasting LDL cholesterol was calculated using the method of Friedewald [11]. Fasting insulin was measured using a TOSOH AIA-II analyzer (TOSOH Corp., South San Francisco, CA).

### 2.5. Preparation of Permeabilized Myofiber Bundles

This technique has been adapted from previously published methods [12]. After an overnight fast, each participant came to the UAB Clinical Research Unit for a skeletal muscle biopsy of the vastus lateralis under local anesthesia. The tissue was cleaned of adipose and connective tissue and a tissue bundle of approximately 20 mg was selected for mitochondrial respirometry experiments. The tissue was transferred to the laboratory on ice in Buffer X containing 50 mM K-MES, 7.23 mM K_2_EGTA, 2.77 mM CaK_2_EGTA, 20 mM imidazole, 0.5 mM dithiothreitol, 20 mM taurine, 5.7 mM ATP, 14.3 mM phosphocreatine, and 6.56 mM MgCl_2_ (pH 7.1, 290 mOsm). Once in the laboratory, the tissue was mechanically dissected into several smaller muscle bundles (of approximately 3–5 mg wet weight). Each bundle was gently separated with a pair of antimagnetic needle-tipped forceps in Buffer X under magnification. Following dissection, each fiber was blotted gently and briefly on an absorbent delicate task wipe. A 1.5 mL tube containing 30 *µ*g/ml saponin in a Buffer X was tared, the fiber was carefully added to the tube, and the tube was reweighed to collect fiber wet weight. Bundles were incubated with saponin on a rotator for 30 min at 4°C. The tissue bundles were washed of saponin for 15 minutes in Buffer Z containing 105 mM K-MES, 30 mM KCl, 1 mM EGTA, 10 mM K_2_HPO_4_, and 5 mM MgCl_2_, 5 *µ*M glutamate, 2 *µ*M malate, and 5.0 mg/ml BSA (pH 7.4, 290 mOsm). Finally, the samples were transferred to Buffer Z containing 20 mM creatine hydrate and 5 *µ*M of blebbistatin for 10 minutes prior to respirometry experiments [13].

### 2.6. Mitochondrial Respiration Measures

High-resolution respirometry experiments were performed using an Oroboros Oxygraph O2K (Oroboros Instruments, Innsbruck, Austria) containing 2 mL of Buffer Z with creatine and blebbistatin, constantly stirred at 37°C. We assessed mitochondrial O_2_ consumption using a FA substrate protocol. Coupled respiration (State 3) was measured using malate (2.5 mM) and palmitoyl carnitine (40 *μ*M) after the addition of a submaximal concentration of ADP (1 mM) [14]. Cytochrome* c* (10 *μ*M) was added to assess mitochondrial membrane integrity after preparation of the myofibers. There was no significant increase in respiration following the addition of cytochrome* c*. Uncoupled respiration (State 4) was induced by the administration of oligomycin (2 *μ*g/mL). Oxygen flux was normalized to the wet weight (ww) of each fiber bundle, a standard method for normalizing respiration rates [15]. The respiratory control ratio (RCR) was calculated as State 3/State 4. The RCR is a commonly used metric for assessing mitochondrial integrity and coupling [16].

### 2.7. Statistical Analyses

All descriptive characteristics are reported as mean ± SD. Pearson correlations were calculated between total and regional fat, RQ, and mitochondrial measures. Partial correlations between mitochondrial measures and leg fat (kg), android fat (kg), and VAT volume (cm^3^) were controlled for the effect of total fat mass (kg). Participants were dichotomized into high body fat and low body fat groups at the median value for percent body fat (39.8%). Differences between groups were assessed using two-tailed* t*-tests for independent samples. An alpha level of *p* < 0.05 was used to determine statistical significance. All statistical analyses were conducted using SPSS Statistics for Macintosh Version 22.0 (IBM Corp., Armonk, NY).

## 3. Results

Demographic, physical, and metabolic characteristics of the study sample are shown in Table 1. BMI ranged from 19.7 to 34.5 kg/m^2^ (mean of 26.9 ± 4.9 kg/m^2^), age ranged from 19 to 44 years (mean of 28.3 ± 7.0 years), and 54% were AA. There were no differences between EA and AA in age, BMI, total body and regional fat measures, or measures of mitochondrial FA oxidation (data not shown).

**Figure 1:**
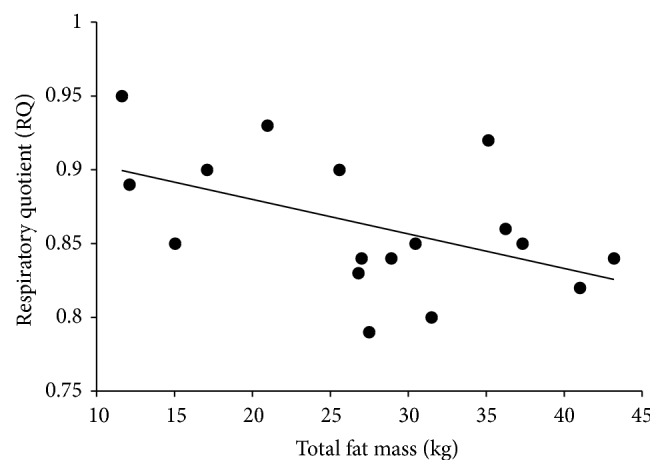
Fasting RQ was negatively associated with total fat mass (*r* = −0.496, *p* = 0.043).

**Figure 2:**
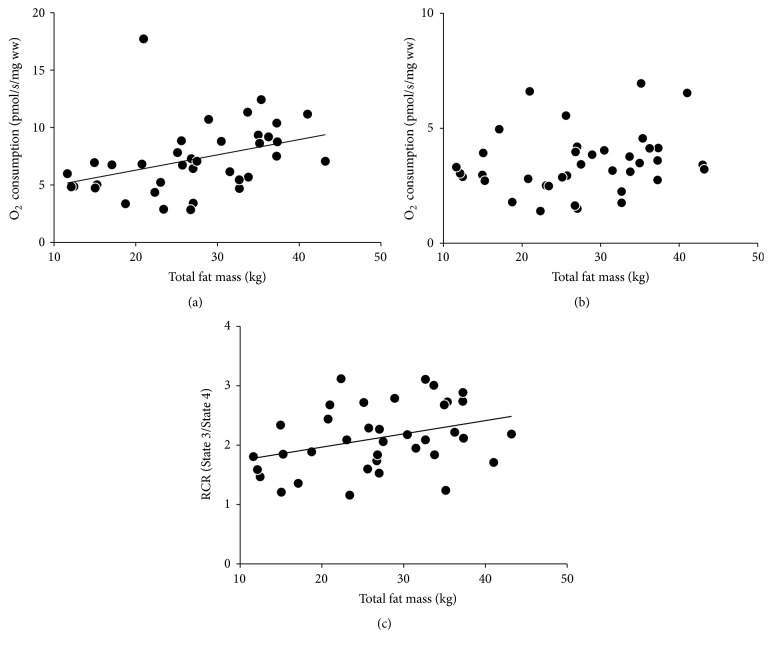
State 3 respiration was positively associated with total fat mass (*r* = 0.379, *p* = 0.021) (a). State 4 respiration was not significantly associated with total fat mass (*r* = 0.216, *p* = 0.193) (b). RCR was positively associated with total fat mass (*r* = 0.348, *p* = 0.035) (c).

**Figure 3:**
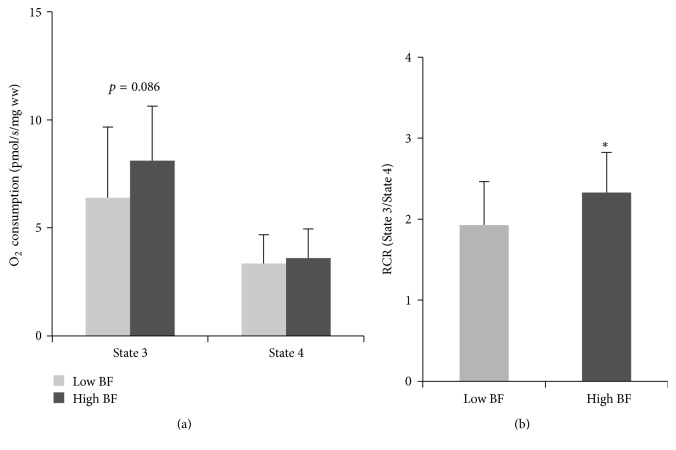
There were no differences in State 3 or State 4 between women with high body fat (BF) and women with low BF (a). RCR was significantly higher in women with high BF (*p* = 0.024) (b). ^*∗*^*p* < 0.05.

In a subset of the cohort for which RQ data were obtained (*n* = 17), there was a negative correlation between total fat mass and resting RQ (*r* = −0.496, *p* = 0.043) (Figure 1). In situ studies revealed significant associations between total fat mass and both State 3 (*r* = 0.379, *p* = 0.021) (Figure 2(a)) and the RCR (*r* = 0.348, *p* = 0.035) (Figure 2(c)). When participants were dichotomized by the median percent body fat, State 3 tended to be higher (*p* = 0.086) (Figure 3(a)) and the RCR was significantly higher (*p* = 0.024) (Figure 3(b)) in those with high body fat (*n* = 18) compared to those with low body fat (*n* = 19).

Android fat and VAT were positively associated with State 3 and RCR (*p* < 0.05) (Table 2). However, after adjusting for total fat mass, no associations between regional fat measures and State 3 or the RCR were observed (Table 2). Leg fat mass was not associated with any measure of mitochondrial function before or after adjusting for total fat mass.

## 4. Discussion

A number of previous investigations have described elevated FA oxidation in obese subjects using* in vivo* methods (e.g., uptake of tracer-labeled intravenous FA) [2, 3]; however whether these findings reflect a shift in substrate-specific oxidative capacity at the level of the skeletal muscle mitochondria had not been examined. The primary finding from this study was that total fat mass was positively associated with FA oxidation capacity assessed in situ in skeletal muscle mitochondria in a sample of premenopausal women. These data suggest that greater FA oxidation in obese subjects is regulated in part by a programmed mitochondrial phenotype that is preserved in situ.

There are a number of possible mechanisms that may mediate the observed positive association between total body fat and in situ fat oxidation. Individuals with obesity are known to have greater intramyocellular lipid content [17], a phenotype that chronically elevates FA substrate availability and promotes substrate competition that may ultimately impair substrate flexibility [8, 9]. Others have suggested that enhanced FA oxidation is a mitochondrial response to a lipid-induced stress due to elevated intramyocellular triglyceride (IMTG), ceramide, and diacylglycerol [18]. Additionally, PGC1*α* is shown to be induced by high-fat feeding and obesity in animal models [6, 7] and plays an important role in regulating mitochondrial FA oxidation [19]. Identifying the precise mitochondrial mechanism responsible for this enhanced FA oxidative capacity may allow a better understanding of the etiology of obesity and the metabolic adaptations it may induce.

In contrast to our findings, lower mitochondrial oxidative capacity has been observed in participants with obesity in other studies. However, these studies did not use FA-derived substrates [20] or observed these effects in cultured tissues [21] and in participants with extreme obesity (BMI > 40 kg/m^2^) [22]. Thus, methodological differences amongst studies may be responsible for differing results. Hulver et al. reported significantly lower skeletal muscle FA oxidation in participants exhibiting extreme obesity (BMI > 40 kg/m^2^) compared to lean controls, but FA oxidation appeared to be modestly increased in a group of overweight and obese subjects (BMI between 25.0 and 34.9 kg/m^2^) (nonsignificant finding, only 8 participants per group) [22]. Taking into account the fact that all participants in our study had a BMI less than 35.0 kg/m^2^, our data in conjunction with the study of Hulver et al. suggests that greater FA oxidation capacity is indeed elevated in obesity but that FA oxidative capacity may become impaired in morbid obesity (BMI > 40 kg/m^2^).

The positive relationship observed between total fat mass and the RCR suggests greater adiposity is associated with a mitochondrial phenotype marked by greater mitochondrial coupling of oxidative phosphorylation. The RCR is an indirect metric for coupling efficiency and we cannot determine cause and effect from this cross-sectional study. However, it is possible that if obese persons display a chronically coupled phenotype, this may promote further fat deposition [23]. Additionally, greater coupling may contribute to an environment prone to high levels of oxidative damage, a characteristic often observed in obesity [24]. Greater mitochondrial coupling promotes the production of reactive oxygen species (ROS) [25, 26] as proton reentry in the mitochondrial matrix independent of ATP synthesis is reduced and substrate utilization is slowed. This coupling phenotype may also promote the accumulation of FA within the mitochondria, which is associated with insulin resistance [27]. The mechanisms that regulate mitochondrial proton leak are induced by FA [28]. Impairment of these mechanisms in the obese state could contribute to oxidative stress and the development of chronic diseases such as type 2 diabetes.

Both metabolic efficiency and FA oxidation are shown to differ between EA and AA individuals [29–33]. However, in the present study, we found no differences in FA oxidation assessed in skeletal muscle mitochondria between EA and AA women (data not shown). Differences in FA oxidation between EA and AA have been ascribed to a difference in FA transport [30]. Because our in situ method bypassed membrane transport, our data support a lack of race difference in FA oxidation at the mitochondrial level and reinforce the importance of FA transport to lower FA oxidation in AA. It will be important in future studies to recruit sufficient numbers of both lean and obese women of both races to clarify if and under what circumstances potential racial differences in mitochondrial FA oxidation exist.

Strengths of this study included robust measures of body composition and the sizeable sample for which human skeletal muscle biopsies were obtained. Mitochondrial function was assessed using permeabilized human myofibers, which allowed us to analyze mitochondrial function within the native cellular system while also preserving interactions with the cytoskeleton and various cellular components. This method may more closely resemble the* in vivo* environment compared to isolated mitochondria. We acknowledge as a limitation that our data may not reflect FA oxidation in other populations, such as men or participants with impaired glucose metabolism. Future studies in these populations are needed to better understand the relationship between FA oxidation and fat mass.

## 5. Conclusions

In conclusion, this study demonstrates that the positive relationship between measures of* in vivo* FA oxidation and obesity is a phenotype preserved at the level of the skeletal muscle mitochondria. Further research is needed to determine the molecular mechanisms responsible for promoting this mitochondrial phenotype and if this increase in FA oxidation is associated with obesity-related comorbidities.

## Figures and Tables

**Table 1:** Participant characteristics.

	*n* = 38
Age (years)	28.3 ± 7.0
Race	17 EA, 21 AA
BMI (kg/m^2^)	26.5 ± 4.3
Total fat mass (kg)	27.5 ± 8.8
Leg fat mass (kg)	11.1 ± 3.5
Android fat mass (kg)	2.0 ± 0.9
VAT volume (cm^3^)^†^	435.2 ± 374.0
Fat-free mass (kg)	45.0 ± 6.1
Total cholesterol (mg/dL)	174.1 ± 30.6
Triglycerides (mg/dL)	78.0 ± 43.4
HDL cholesterol (mg/dL)	62.6 ± 14.4
LDL cholesterol (mg/dL)	95.8 ± 23.3
Free fatty acids (mEq/L)^‡^	0.6 ± 0.2
Fasting glucose (mg/dL)^†^	88.6 ± 7.5
Fasting insulin (*μ*IU/mL)^†^	8.7 ± 3.3
Fasting RQ (VCO_2_/VO_2_)^#^	0.86 ± 0.05
State 3 (pmol/s/mg)^†^	7.2 ± 3.0
State 4 (pmol/s/mg)	3.5 ± 1.3
RCR (State 3/State 4)^†^	2.1 ± 0.6

^†^ *n* = 37. ^‡^*n* = 35. ^#^*n* = 17. AA, African-American; BMI, body mass index; EA, European-American; RCR, respiratory control ratio; RQ, respiratory quotient; VAT, visceral adipose tissue.

**Table 2:** Pearson correlation coefficient (*p* value) and partial correlation coefficient adjust for total fat (kg) (*p* value). ^*∗*^*p* < 0.05.

	State 3	State 4	RCR	State 3 (adj.)	State 4 (adj.)	RCR (adj.)
Total fat mass (kg)	0.379 (**0.02**1^*∗*^)	0.216 (0.193)	0.348 (**0.03**5^*∗*^)	—	—	
Leg fat mass (kg)	0.256 (0.126)	0.147 (0.379)	0.286 (0.086)	−0.208 (0.232)	−0.108 (0.537)	−0.097 (0.578)
Android fat mass (kg)	0.440 (**0.00**6^*∗*^)	0.253 (0.125)	0.360 (**0.02**8^**∗**^)	0.283 (0.099)	0.157 (0.314)	0.110 (0.530)
VAT volume (cm^3^)	0.424 (**0.01**0^*∗*^)	0.215 (0.201)	0.399 (**0.01**6^*∗*^)	0.235 (0.175)	0.091 (0.605)	0.158 (0.363)

RCR, respiratory control ratio; VAT, visceral adipose tissue.
